# Unveiling the stealthy tactics: mycoplasma’s immune evasion strategies

**DOI:** 10.3389/fcimb.2023.1247182

**Published:** 2023-08-31

**Authors:** Jingyun Wang, Keying Liang, Li Chen, Xiaoling Su, Daoyong Liao, Jianwei Yu, Jun He

**Affiliations:** ^1^The Affiliated Nanhua Hospital, Department of Clinical Laboratory, Hengyang Medical School, University of South China, Hengyang, Hunan, China; ^2^Department of Public Health Laboratory Sciences, School of Public Health, Hengyang Medical School, University of South China, Hengyang, Hunan, China

**Keywords:** mycoplasma, immune evasion, antigen variation, phagocytosis, neutrophil extracellular traps

## Abstract

Mycoplasmas, the smallest known self-replicating organisms, possess a simple structure, lack a cell wall, and have limited metabolic pathways. They are responsible for causing acute or chronic infections in humans and animals, with a significant number of species exhibiting pathogenicity. Although the innate and adaptive immune responses can effectively combat this pathogen, mycoplasmas are capable of persisting in the host, indicating that the immune system fails to eliminate them completely. Recent studies have shed light on the intricate and sophisticated defense mechanisms developed by mycoplasmas during their long-term co-evolution with the host. These evasion strategies encompass various tactics, including invasion, biofilm formation, and modulation of immune responses, such as inhibition of immune cell activity, suppression of immune cell function, and resistance against immune molecules. Additionally, antigen variation and molecular mimicry are also crucial immune evasion strategies. This review comprehensively summarizes the evasion mechanisms employed by mycoplasmas, providing valuable insights into the pathogenesis of mycoplasma infections.

## 1 Introduction

Mycoplasmas exhibit a wide distribution in their natural habitat, acting as parasites in various organisms, including humans, mammals, reptiles, fish, arthropods, and plants (Razin et al., 1998). These smallest known self-replicating prokaryotic microorganisms consist of a plasma membrane, ribosomes, circular double-stranded chromosomes, and lack a cell wall. Due to their restricted biosynthesis capacity, most mycoplasmas function as parasites displaying stringent host and tissue specificities (Rottem, 2003). With high prevalence, mycoplasmas exert a significant economic influence on healthcare and biomedical research and as infectious agents affecting cattle, swine, sheep, and other agricultural animals. For example, *Mycoplasma genitalium*, which invades the human genitourinary tract, is a common cause of sexually transmitted infections (STIs), including male urethritis and cervicitis, endometritis, pelvic inflammatory disease, and possible preterm birth, tubal infertility, and ectopic pregnancy in women (McGowin and Totten, 2017). Besides causing severe lower respiratory tract disease and mild upper respiratory tract symptoms in humans, *Mycoplasma pneumoniae* can also cause widespread extrapulmonary infections and post-infectious events (Atkinson et al., 2008). *Mycoplasma gallisepticum*, *Mycoplasma bovis*, *Mycoplasma hyopneumoniae*, etc. can also bring economic losses to animal husbandry (Dudek et al., 2020; Leal Zimmer et al., 2020; Feberwee et al., 2022). In short, it is necessary to understand the physiological characteristics and pathogenic mechanism of mycoplasma.

During the parasitism cycle, mycoplasma will adhere to the host cell, which is closely related to adhesin, accessory protein, and potential moonlight protein. Certain inherent constituents of the cell membrane of mycoplasma, including lipids and membrane lipoproteins, can elicit inflammatory reactions and induce tissue injury through diverse mechanisms (Yiwen et al., 2021). Other pathogenic materials, such as metabolic enzymes, phosphatase, cytotoxic nucleases, etc., are also considered essential mycoplasma pathogens (Xie et al., 2021). At the same time, mycoplasma utilizes nutrients from host cells and secrete substantial quantities of metabolites, such as hydrogen peroxide (H_2_O_2_) and hydrogen sulfide (H_2_S) (Großhennig et al., 2016; Blötz and Stülke, 2017). All of these could induce cellular toxicity and damage the tissues.

**Figure 1:**
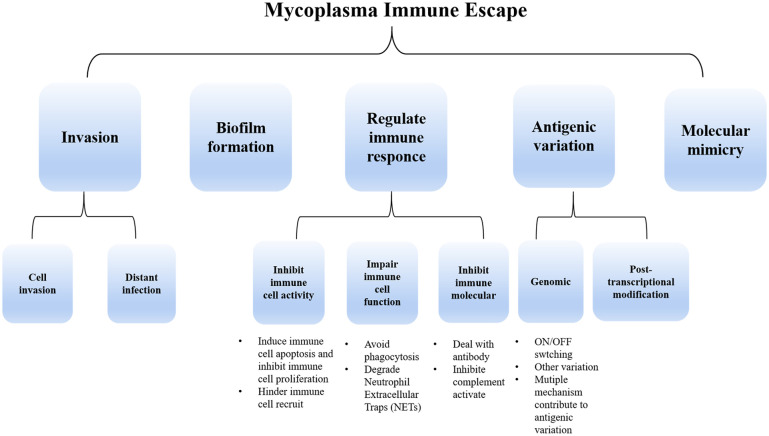
The primary immune escape strategies employed by mycoplasma include invasion, biofilm formation, and regulation of immune response (through the suppression of immune cell activity and function as well as immunomodulatory molecule inhibition). Additionally, mycoplasma employs antigenic variation strategies both at the genomic level and post-transcriptionally. Molecular mimicry is also involved in mycoplasma immune escape.

The host immune system serves as a defense mechanism against invading microbes, comprising mainly innate immune and adaptive immunity. The innate immune response involves the participation of epithelial cells located in mucosal surfaces and phagocytic cells that are recruited from the blood, which include granulocytes, monocytes, and macrophages (Kogut et al., 2020). The pattern recognition receptors (PRRs) in the innate immune cell can detect microbial presence, including mycoplasma. Lipoproteins or lipopeptides, synthesized by mycoplasma, may be acted as microbial-associated molecular patterns (MAMP), which can be recognized by innate immune cells and initiate immunization (Zuo et al., 2009). Macrophages can produce defense proteins or directly phagocyte pathogens, facilitating the eradication of pathogens (Maes et al., 2021). Neutrophils can release neutrophil extracellular traps (NETs) to digest pathogens. Similarly, during the adaptive immune response, B lymphocyte cells generate targeted antibodies, while T lymphocytes contribute to triggering diverse factors to activate other immune cells. Different types of antigen-presenting cells and T-help cells (Th1/Th2) also have a significant role in the defense mechanism against the invasion of mycoplasma. However, mycoplasmas have the capability to persist and survive within the host for extended periods. This prolonged resistance to the host immune elimination is primarily attributed to the evolution of sophisticated strategies employed by mycoplasmas. While recent advancements in molecular biology, genomics, and proteomics have facilitated the study of the limited mycoplasma genome, the factors underlying their adhesion, virulence, pathogenesis, and immune evasion abilities still require further elucidation. Therefore, the review summarizes the critical immune evasion mechanisms employed by mycoplasmas, including invasion, biofilm formation, and modulation of immune responses, which include the inhibition of immune cell activity, suppression of immune cell function such as avoid phagocytosis, degradation NETs, resistance against immune molecules, antigen variation, and molecular mimicry. (**Figure 1**)

## 2 Invasion

### 2.1 Cell invasion

Mycoplasma utilizes cell invasion as an effective tactic to evade the immune system. By residing within host cells, mycoplasma can effectively evade immune cells and immune effector molecules, thus evading recognition and destruction. Previous research erroneously characterized mycoplasma as an extracellular pathogen. However, with the advancement of experimental tools like laser confocal and electron microscopy, it has become evident that mycoplasma can exist within non-phagocytic eukaryotic cells. The first mycoplasma reported to invade cells was *Mycoplasma penetrans*, which was isolated from the genitourinary tract of immunodeficiency (AIDS) patients. Since then, other mycoplasmas such as *M. pneumonia*, *M. genitalium*, *M. bovis, Mycoplasma agalactiae* (Meseguer et al., 2003; McGowin et al., 2009; van der Merwe et al., 2010; Hegde et al., 2014; Bürki et al., 2015a), etc. were also discovered to possess the ability to invade cells. And most of these mycoplasmas colonize in the cytoplasm and perinuclear sites within the host cells.

The invasion process is highly intricate and necessitates comprehensive elucidation. Adhesion, receptor binding, signal pathway transduction, skeleton protein rearrangement, membrane fusion, and endocytosis are potential contributing factors to mycoplasma invasion. Adhesion appears to be the primary and crucial step for mycoplasma invasion. Recent research indicates that bacterial invasion is facilitated by the ability to bind to sulfated polysaccharides or fibronectin (Fn) (Yavlovich et al., 2001; Rottem, 2003). *M. pneumoniae* is one of the most typical mycoplasmas that can bind sulfated polysaccharides (Athamna et al., 1996). Transcriptomic analysis of *Mycoplasma hominis* infected cells has demonstrated a significant upregulation of genes related to the extracellular matrix (ECM) receptor interaction pathway and phagosome-related integrins (Hopfe et al., 2013). Fn, a component of ECM, can interact with integrin, which is known to induce cytoskeletal rearrangements in eukaryotic cells to facilitate pathogen uptake (Henderson et al., 2011; Hauck et al., 2012; Harvey et al., 2019). *M. hyopneumoniae* can link with integrin β1 by Fn and promotes mycoplasma uptake as a vesicle coated with caveola and clathrin. Co-incubating with β1 integrin antibody can inhibit the adhesion of *M. hyopneumoniae* to host cells (Raymond et al., 2018). The fructose-1,6-bisphosphate aldolase (FBA) encoded by the core gene of *M. pneumoniae* (Yu et al., 2018a) and *M. bovis* (Huang et al., 2019) has been found to mediate adhesion with Fn. *M. hyopneumoniae* recombinant elongation factor thermo unstable (rEF-Tu) can bind with Fn in a dose-dependent and physiological manner *in vitro* (Yu et al., 2018b). However, whether the adhesion contributes to internalization remains to be investigated.

**Figure 2:**
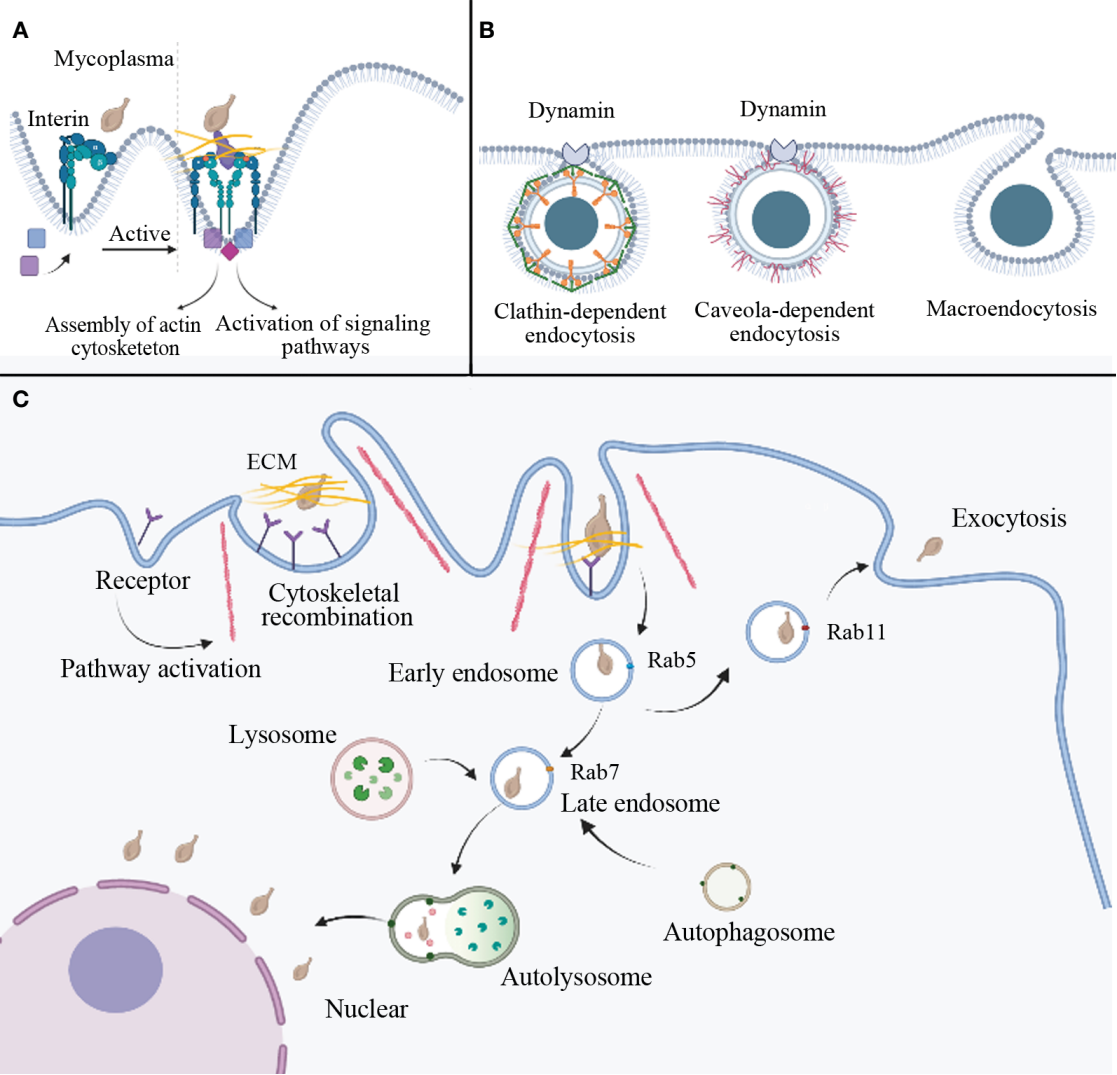
Potential mechanisms of mycoplasma internalization. **(A)** Mycoplasma can interact with host cell receptors to mediate endocytosis. For example, when mycoplasma interacts with the ECM and integrin, it activates a cellular pathway that induces cytoskeletal reorganization and membrane invagination to prepare for endocytosis. **(B)** Three pathogen endocytosis pathways are mediated by clathrin, caveolae, and macropinocytosis. **(C)** The possible process of mycoplasma internalization involves vesicle formation. Mycoplasma enters the cell via vesicle wrapping, forming an early endosome marked by Rab5, followed by a late endosome marked by Rab7. After fusion with autophagic and lysosomal bodies, autolysosomes are formed, which can degrade external pathogens. However, some mycoplasma may escape lysosomal degradation and settle in the perinuclear region. In addition to the above pathways, mycoplasma-wrapped vesicles can also form recycling endosome, mainly marked by Rab11, and infect surrounding cells via exocytosis. ECM: extracellular matrix.

The binding of integrins to ECM ligands triggers signaling pathways that recruit scaffolding/adapter molecules, kinases, and phosphatases to form protein complexes. These complexes play a role in modulating cellular cytoskeletal dynamics and regulating various cellular activities (Moreno-Layseca et al., 2019). (**Figure 2A**) The invasiveness of *M. penetrans* can be attenuated by using cytochalasin D to inhibite the assembly of actin filaments in HeLa cells. Additionally, complete loss of invasiveness can be achieved by disrupting the microtubule structure through the use of vinblastine or paclitaxel. These observations suggest that mycoplasma may enter HeLa cells by reorganizing cytoskeletal components. Moreover, in this experiment, the activation of protein kinase C was observed in host cells following mycoplasma infection, indicating a potential association between mycoplasma invasion and protein kinase activation (Borovsky et al., 1998). Conversely, the penetration of *M. gallisepticum* into chicken embryo fibroblasts was found to be impeded by the microtubule inhibitor nocodazole but unaffected by cytochalasin D (Winner et al., 2000), indicating that *M. gallisepticum* may employ an alternative invasion mechanism. In summary, the invasion of mycoplasma involves the rearrangement of the eukaryotic cell cytoskeleton, and the underlying mechanism may be mediated through host receptor-mediated signal transduction, leading to alterations in cellular actin or microtubules.

The mechanisms of virus and bacterial internalization into host cells primarily involve fusion and endocytosis pathways. Endocytic pathways for animal viruses entering host cells include macropinocytosis, clathrin-dependent endocytosis, and caveola-dependent endocytosis. (**Figure 2B**) Rab proteins participate in intracellular and aspects of extracellular protein transport (Nishiumi et al., 2017). Internalization of *M. bovis* can be disrupted by the inhibitor of clathrin-dependent endocytosis (dansylcadaverine) and cholesterol-mediated endocytosis (simvastatin), showing that *M. bovis* enters bovine synovial cells mainly through the route mentioned above (Nishi et al., 2021). *M. hyopneumoniae* cells are internalized through clathrin-mediated and caveolae-mediated endocytosis and transported intracellularly along the entire endocytic pathway (Raymond et al., 2018). Upon entering HeLa cells, *Ureaplasma parvum* forms early endosomes marked by Rab5 within clathrin-coated vesicles. The encapsulated mycoplasma can evade immune effector molecules. As the vesicles mature, late endosomes marked by Rab7 appear. Fusion of late endosomes with lysosomes leads to mycoplasma degradation by endocytic digestive enzymes. However, a small number of mycoplasmas can escape degradation and colonize the cytoplasm and perinuclear region, even infecting surrounding cells through exocytosis (Nishiumi et al., 2017). (**Figure 2C**) Mycoplasma infection inhibits the autophagic degradation of LC3-II and p62. Upregulation of Rab7 and inhibition of autophagic degradation synergistically contribute to intracellular mycoplasma accumulation (Hu et al., 2014). *M. hyopneumoniae* employs the JNK and Akt signaling pathways to induce incomplete autophagy in porcine alveolar macrophages. Incomplete autophagy serves as a defense mechanism, impeding the entry of *M. hyopneumoniae* into lysosomes and preventing its degradation, allowing the proliferation of *M. hyopneumoniae* within porcine alveolar macrophages (Wen et al., 2022). Lipid rafts also seem to be involved in mycoplasma invasion, it possibly associated with caveola-dependent endocytosis (Quest et al., 2004). Lipid rafts are specialized lipid microdomains abundant in sphingolipids, cholesterol, and glycosylphosphatidylinositol-anchored proteins. Depletion or sequestration of cholesterol will destroy the lipid rafts. Studies have shown that *M. gallisepticum* displays lower invasiveness when infecting cholesterol-depleted HeLa cells (Fürnkranz et al., 2013), possibly due to protein and lipid dispersion caused by cholesterol deficiency (Mañes et al., 2003).

In a word, the mechanism of mycoplasma cell invasion can be succinctly delineated as follows: mycoplasma engages in receptor binding with host cells, thereby eliciting signal transduction cascades, prompting cytoskeletal rearrangements, resulting in cellular membrane invagination and the subsequent formation of endocytic vesicles. Mycoplasma is ensheathed within these vesicles and subsequently undergoes fusion with mature vesicles, enabling it entry into lysosomes. Some mycoplasma species employ strategies to evade lysosomal destruction, enabling them to escape into the cytoplasm or localize in the perinuclear region. Moreover, the vesicles that enshroud mycoplasma during the maturation process exhibit a cyclic nature, permitting their exocytosis and subsequent dissemination, thereby facilitating the infection of neighboring cells.

### 2.2 Distant infection

Indeed, cell invasion is an important strategy for mycoplasma to evade the immune system. In fact, mycoplasma invasion is not limited to the primary site of infection. Mycoplasma have the ability to disrupt intercellular connections and the integrity of epithelial cell barriers, thereby expanding infection. *M. fermentum*, *M. penetranum*, and *Mycoplasma piriformis* can hydrolyze arginine, ferment glucose, and invade eukaryotic cells. These mycoplasmas are also considered potential cofactors that accelerate the progression of HIV infection due to their ability to adhere to and destroy epithelial cells (Blanchard and Montagnier, 1994). *M. hyopneumoniae* has the ability to adhere to pig bronchial epithelial cells (PBECs) and impair mucociliary function. Notably, it can translocate to the basolateral chamber via the paracellular route rather than the transcellular pathway. This translocation is facilitated by the reversible disruption of tight junctions (TJs), leading to increased permeability of the epithelial barrier (Wang et al., 2020). Through the aforementioned pathways, mycoplasma can breach the immune barrier of epithelial cells, enabling further infection.

**Figure 3:**
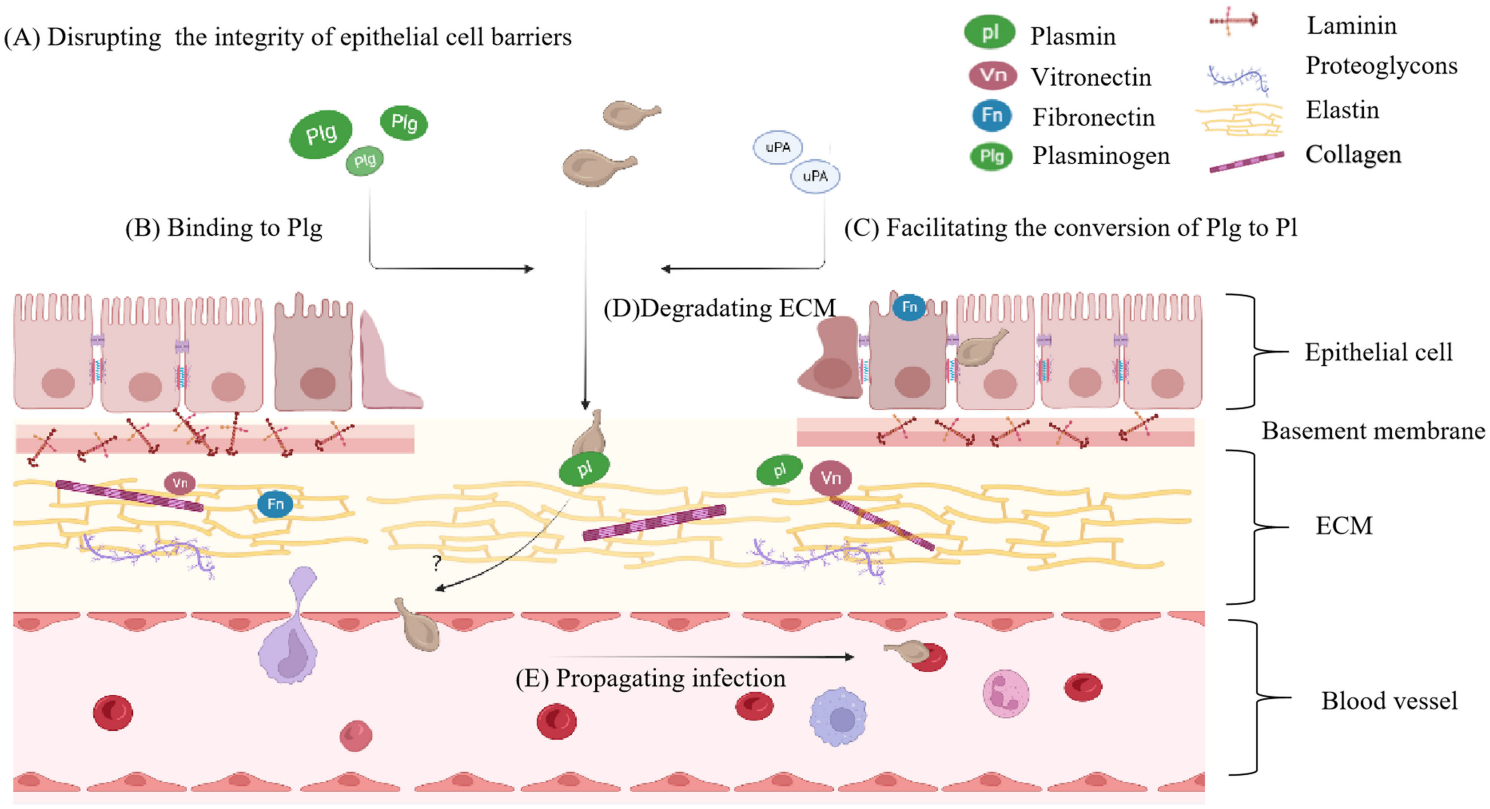
The principal phases of distant invasion by mycoplasma. **(A)** The principal phases of distant tissue invasion by mycoplasma begin with disrupting intercellular connections and the integrity of epithelial cell barriers. **(B)** Mycoplasma will bind to Plg, then **(C)** hijack host activators or employ intrinsic activating proteins to activate the Plg transform into Pl. **(D)** Pl degarde ECM component. **(E)** As the ECM breaks down, mycoplasma is able to propagate the infection. Plg, plasminogen; Pl, plasmin.

By hijacking plasminogen (Plg) activators or expressing Plg receptors on their surface, numerous invasive bacteria have the ability to disperse from their initial point of colonization and enter distant tissue sites via the fibrinolytic system (Wang et al., 2022). It is closely associated with Plg and its conversion to plasmin (Pl). Pl, a proteolytic enzyme with broad substrate specificity, can activate latent matrix metalloproteinases to hydrolyze the ECM compenent including collagen, Fn, Laminnin (Ln) etc.(Singh et al., 2012), enabling mycoplasmas to spread through connective tissues, evade inflammatory responses, and expand the site of infection (Yavlovich et al., 2001) (**Figure 3**). *M. fermentans* can bind to Plg and hijack host activators to convert it into Pl, modifying its surface proteins and promoting internalization (Yavlovich et al., 2004). In the presence of a urokinase-type plasminogen activator (uPA), mycoplasma invasion becomes more pronounced (Yavlovich et al., 2001). In recent years, studies have shown that the protein or enzyme existing in mycoplasma itself can activate Plg and degrade ECM, such as Glyceraldehyde-3-phosphate dehydrogenase (GAPDH) from *M. hyopneumoniae* (Wang et al., 2023). *M. pneumoniae* is not limited to the respiratory system but can invade vascular endothelial cells and lead to encephalitis (Rhodes et al., 2011). *Mycoplasma hyorhinis*, a frequent inhabitant of the porcine respiratory tract, can cause serofibrinous inflammation of serous body cavities and joints in piglets, including polyserositis, arthritis, eustachitis, otitis, conjunctivitis, meningoencephalitis, and pneumonia (Babajani et al., 2022). Numerous studies have shown the interaction between mycoplasmas, ECM, and Pl. Recombinant *M. hyorhinis* enolase (rEno) has been shown to bind to Fn and Plg, exploiting host activators to generate Pl. Substitution of lysine residues with leucine at the C-terminus of rEno results in reduced binding capacity, indicating that the two lysine residues at the C-terminus are critical binding sites for its multifunctional binding activity (Wang et al., 2022). The immunogenic proteins pyruvate dehydrogenase E1 alpha subunit and pyruvate dehydrogenase E1 beta subunit of *M. gallisepticum* can bind to the Plg, facilitating bacterial adhesion, colonization, transmission, and affecting the virulence of *M. gallisepticum* (Fürnkranz et al., 2013; Qi et al., 2018).

## 3 Biofilm formation

The biofilm can be defined as a community of microorganisms attached to a surface or embedded in the extracellular matrix (Roy et al., 2018). They enhance the resistance of pathogens to immune surveillance and antimicrobial agents, leading to chronic infections. Compared to planktonic cells, biofilms exhibit greater resilience to external stressors such as heat and dryness (McAuliffe et al., 2006; Bürki et al., 2015b). The increased resistance of biofilms to antibiotics and dry conditions can be attributed to metabolic changes and distinctive protein expression profiles (Chen et al., 2018). While some mycoplasma biofilms show similar susceptibility to antibiotics as planktonic cells, most mycoplasma biofilms display higher resistance to antibiotics. For example, *M. hyopneumoniae* biofilms can survive antibiotic concentrations that are 10-fold higher than the minimum inhibitory concentration (MIC) for planktonic cells (Tassew et al., 2017). The ability of biofilm to withstand antimicrobial agents and evade immune surveillance is partially attributed to the production of extracellular polymeric substances (EPS), which consist of lipids, proteins, DNA, and exopolysaccharides. EPS acts as a physical barrier, inhibiting the diffusion of drugs, antibodies, and immune cells into biofilms (Simmons et al., 2007; Versey et al., 2021). Biofilms can also modulate the adaptive immune responses of the host by altering pathogen sensitivity to complement and antimicrobial peptides released by immune cells (Jahan et al., 2022). Furthermore, biofilms can capture inflammatory products and mechanically prevent the activation of inflammatory signaling pathways (García-Castillo et al., 2008; Kallapur et al., 2011). Several studies have found a positive correlation between the virulence of mycoplasma strains and their ability to form biofilms (Wu et al., 2022).

It has been observed that several mycoplasma, which colonize the human respiratory and urogenital tracts, have the ability to form biofilms (García-Castillo et al., 2008; Feng et al., 2021). In general, the process of biofilm formation of bacteria involves four significant steps: (i) reversible initial adhesion of cells to a surface; (ii) irreversible early development of the biofilm structure; (iii) maturation of the fully developed biofilm; and (iv) dispersion of cells into the planktonic state (Sauer, 2003). While the dynamic process of mycoplasma biofilm formation may not have been extensively studied, membrane lipoproteins play a crucial role in mediating adhesion within bacterial communities. In the case of *M. pneumoniae*, the interaction between its tip organelle and cell surface sialylated oligosaccharide receptors serves as the initial step in biofilm formation. Specific antibodies against the P1 protein have been shown to inhibit the formation of *M. pneumoniae* biofilms (Kornspan et al., 2011). Sliding mismatches in the Vsa repeat tandems can alter the size of the Vsa protein, thereby affecting the ability to form biofilms (Simmons et al., 2007). Mycoplasma strains producing short Vsa proteins (5 or less) can attach to surfaces such as glass and polystyrene to form biofilms, whereas those with long Vsa proteins tend to remain in a planktonic state (Simmons and Dybvig, 2009; Feng et al., 2015).

In addition, polysaccharides can also affect the formation of biofilms. In the case of mycoplasma strains with EPS-I mutants, we have observed that they are able to form biofilms regardless of the presence of a lengthy Vsa protein. The heightened capacity of the mutants to create biofilms is attributed to their excessive production of EPS-II. And it is predicted to possess a terminal N-acetylglucosamine (GlcNAc) residue based on its lectin-binding properties (Simmons and Dybvig, 2009). While GlcNAc, as a polysaccharide component in the intercellular matrix, is beneficial to forming staphylococcal biofilms (Sultan et al., 2021), we speculate that it may also play an important role in mycoplasma biofilm formation. In *M. galliscepticum* mutants with disrupted genes, including ManB (MGA_0358), an ABC transporter permease (MGA_0689), and an ABC transporter ATP-binding protein (MGA_0655), which are involved in the production of extracellular polysaccharides, the ability to form biofilms showed varying levels of decline compared to the wild type (Wang et al., 2017). This suggests that adhesins and the production of mycoplasma polysaccharides are conducive to biofilm formation.

Besides the factors above, several external factors may also impinge upon the formation of biofilms. For instance, the supplementation of peroxidase appears to promote *M. pneumoniae* biofilm formation, and the underlying mechanisms remain unclear (Simmons and Dybvig, 2015). Compared to a medium supplemented with arginine, adding thymidine could significantly enhance the yield of *M. hominis* biofilm formation (Evsyutina et al., 2022). It is presumably due to the growth of mycoplasma biofilm is influenced by varying energy sources. Glucose at concentrations ranging from 0.5% to 5% can facilitate *M. gallisepticum* biofilm formation while adding EDTA or a glucose or sucrose concentration exceeding 5% can inhibit biofilm formation (Chen et al., 2012). The formation of mycoplasma biofilm contributes to immune evasion and can cause chronic and persistent infections. Consequently, further research on the molecular mechanisms underlying the formation and regulation of mycoplasma biofilm is warranted.

## 4 Modulate immune response

### 4.1 Inhibit the activity of immune cells

#### 4.1.1 Induce immune cell apoptosis and inhibit immune cell proliferation

Understanding the immune evasion mechanisms employed by mycoplasma requires a comprehensive investigation of the interaction between mycoplasma and immune cells. The spleen and thymus, serving as primary organs of immunity, play a preeminent role in generating and maturing immune cells. *M. gallisepticum* can trigger changes in mitochondrial dynamics in spleen and thymus cells, leading to mitochondrial damage and ultimately inducing cell death (Hu et al., 2021). Mycoplasma can also directly induce apoptosis of vital immune cells to reduce the clearance ability of the host. Lipoproteins of the mycoplasma could induce apoptosis in monocyte (e.g. HL-60 and THP-1) and lymphocyte (e.g. MOLT-4) cell lines and regulate adaptive immunity of both B cells and T cells to facilitate evasion of the host immune response. It may be closely related to the activation of p38 MAPK, apoptosis signal-regulated kinase (ASK1), and NF-kB pathway in host cells (Christodoulides et al., 2018). *M. bovis* can enhance neutrophil apoptosis, stimulate the production of proinflammatory cytokines (e.g. IL-12 and TNF-α), inhibit the production of nitric oxide, increase elastase release and suppress the ability of neutrophils to destroy mycoplasmas (Jimbo et al., 2017).

**Figure 4:**
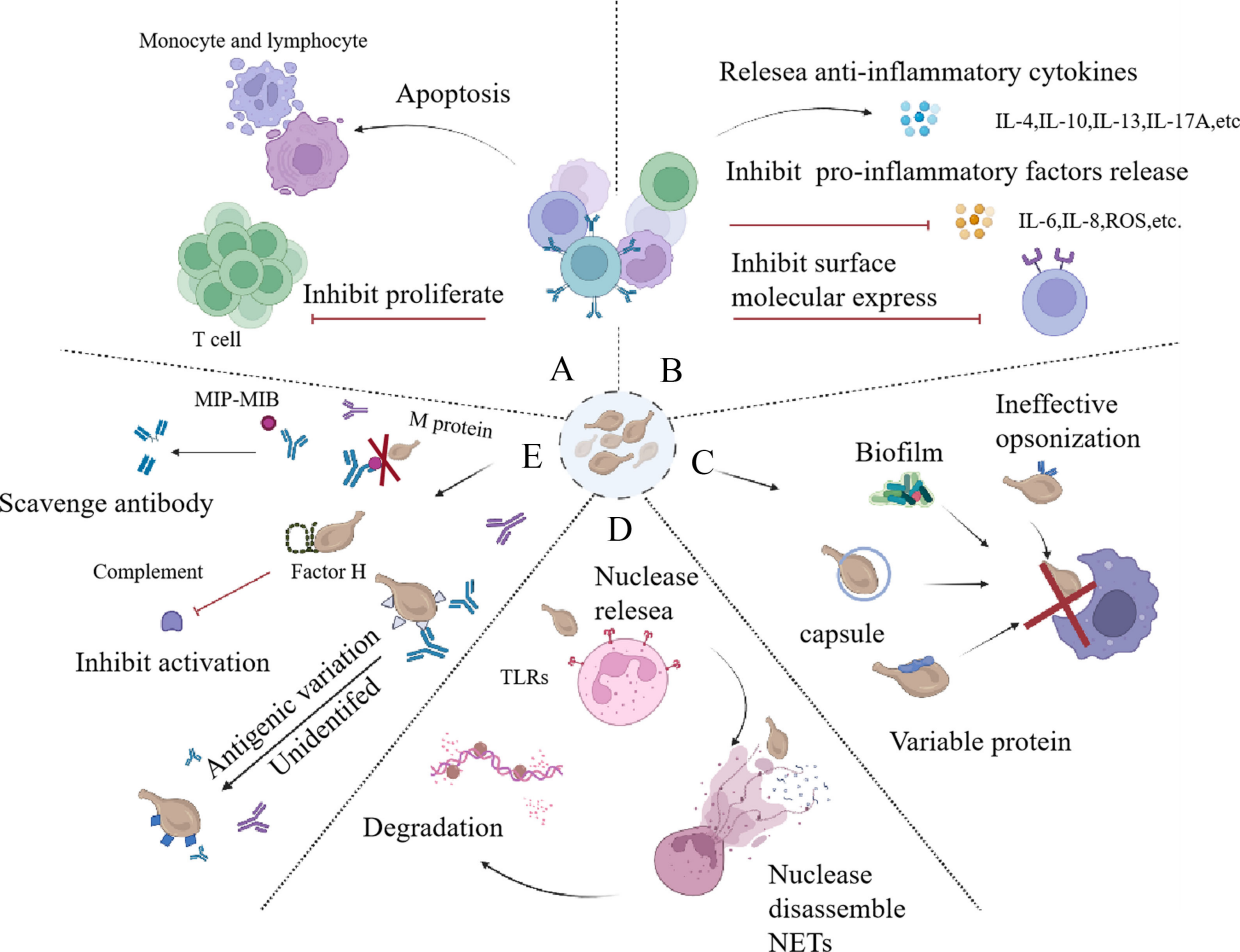
Strategies employed by mycoplasma to counteract immune responce. **(A)** The inhibition of immune cell activity occurs primarily through the promotion of immune cell apoptosis and the suppression of immune cell proliferation. **(B)** The immune cell recruitment is hindered through the stimulation of the release of anti-inflammatory factors, suppression of the release of pro-inflammatory factors, and restriction of the expression of surface molecules. **(C)** Mycoplasma can avoid phagocytosis, which is mainly relay on the ineffective opopsonin, biofilm, capsule, and its surface variable protein. **(D)** Mycoplasma can release nuclesea to degradate NETs to escape immune responce. **(E)** Mycoplasma can combat with immune effector molecules, including scavenging antibody by releasing MIB, MIP, using protein M to prevent the formation of antigen-antibody complexes, utilizing antigenic variation to evade antibody-mediated killing, and binding factor H to escape complement-mediated killing. NETs, neutrophil extracellular traps; MIB, mycoplasma Ig-binding protein; MIP, mycoplasma Ig protease.

*M. bovis* can inhibit T cell proliferation in chronic infections, causing T cell exhausted (Maunsell and Chase, 2019). *M. gallisepticum* exhibits a systemic immunosuppressive impact, diminishing the mitogenic activity in splenic lymphocytes (Browning et al., 2011). *Mycoplasma mycoides ssp mycoides* biotype small colony-secreted components can induce apoptotic cell death in bovine leukocytes. By triggering host cell cytotoxicity, they may develop an effective method for escaping the bovine immune response (Dedieu et al., 2005). Chen X, et al. provide a distinctively pellucid expression profile mainly belonging to the isolated BALF T cells in severe mycoplasma pneumoniae pneumonia (MPP) children demonstrating that in the inflammatory airway, overactivated T cells were exhausted and on the verge of apoptotic progress (Chen et al., 2021). In general, mycoplasma displays a huge capacity to circumvent host immunity mechanisms by inducing apoptosis, and inhibiting cell proliferation (**Figure 4A**). Consequently, these may facilitate the organism’s ability to evade effective immune responses.

#### 4.1.2 Hinder immune cell recruit

Mycoplasma has the ability to hinder the recruitment of immune cells by interfering with the inflammatory response (**Figure 4B**). Lipoproteins in mycoplasma play a role in modulating the host immune response. These Lipid Associated Membrane Proteins (LAMPs) can promote the nuclear translocation of the transcription factor nuclear factor erythroid 2-related factor 2 (Nrf2). Nrf2 activation leads to increased expression of heme oxygenase-1 (HO-1), an enzyme involved in cellular defense against oxidative stress. By upregulating HO-1, LAMPs can negatively regulate the release of pro-inflammatory molecules in THP-1, such as interleukin-6 (IL-6), interleukin-8 (IL-8), reactive oxygen species (ROS), nitric oxide (NO), and prostaglandin E2 (PGE2). Inhibiting the release of these molecules helps mycoplasma evade the immune system and dampen the overall inflammatory response (Hu et al., 2017; Chernov et al., 2018). These molecules are important mediators of the inflammatory response. Two representatives of the mollicutes can produce LAMPs to modulate inflammatory, which are *M. pneumoniae* and *M. genitalium* (He et al., 2015; Hu et al., 2017). Other mycoplasma species, such as *Ureaplasma urealyticum* and *M. hominis*, can stimulate the release of anti-inflammatory cytokines IL-10 and IL-13 (Noda-Nicolau et al., 2016). With *M. pneumoniae* infection, frequent and concentrated sensitization induced exacerbation of lung inflammation immunologically and pathologically and evoked intrapulmonary IL-17A and IL-10 production (Kurata et al., 2014). *M. pneumoniae* P1 protein can promote IL-4 release from rodent mast cells (Hoek et al., 2005). Mycoplasma can also inhibit the recruitment of immune cells by down-regulating the expression of cell surface molecules (such as CD62L), which helps bacteria evade immune clearance (Jimbo et al., 2017). In conclusion, mycoplasma can attenuate immune response by inhibiting immune cell activity.

### 4.2 Impair immune cell function

#### 4.2.1 Avoid phagocytosis

Neutrophils and macrophages constitute the first line of defense against pathogenic microorganisms. Alannah S. Deeney et al. detected *M. hyopneumoniae* labeled with a green fluorescent protein (GFP) within macrophages. Over time, the intracellular *M. hyopneumoniae* in macrophages increased gradually, as observed through confocal microscopy. Interestingly, neither porcine serum complement nor convalescent serum could enhance the phagocytosis of porcine *M. pneumoniae*, suggesting the ability of porcine *M. hyopneumoniae* to evade extensive phagocytosis by macrophages (Deeney et al., 2019). In necrotic and inflamed bovine lung tissue, a large number of intact *M. bovis* can be observed alongside numerous neutrophils and macrophages, indicating that phagocytes are unable to completely eliminate *M. bovis*. Effective phagocytosis of *M. bovis* by bovine neutrophils and macrophages depends on opsonization (Hermeyer et al., 2012). Opsonins are also crucial factors involved in the immune response. Bacteriological and immunohistochemical examination of *M. bovis* infected issue demonstrated the persistence of *M. bovis* and its ability to evade specific immune responses. This is attributed to the downregulation of antigen-presenting mechanisms and inadequate humoral immune responses, with IgG1 dominating the humoral immune response. In comparison to the IgG2 antibody, IgG1 functions as a relatively ineffective opsonin in the immune response. This immune evasion mechanism enables *M. bovis* to persist in the host and evade targeted immune responses (Hermeyer et al., 2012).

According to the information above, mycoplasma can avoid phagocytosis by inducing apoptosis of phagocytic immune cells. Mycoplasma’s substances, such as lipoproteins and the capsule on the surface, can also play a role in avoiding phagocytosis. *M. pneumoniae* can produce a Vsa family of anti-phagocytic lipoproteins with variable phases and sizes and ultimately avoid being phagocytized (Shaw et al., 2012). It may be due to the mechanism that the long Vsa protein can inhibit the recognition of mycoplasma surface proteins with phagocyte cell surface receptors. The capsular polysaccharide of *M. pneumoniae* is also a common anti-phagocytic factor, which can reduce the combination of *M. pneumoniae* with alveolar macrophages (Shaw et al., 2012; Shaw et al., 2013). These findings collectively suggest that mycoplasma possesses the ability to escape phagocytosis. (**Figure 4C**)

#### 4.2.2 Degrade neutrophil extracellular traps (NETs)

NETs are intricate structures consisting of DNA, antimicrobial peptides, and various enzymes that are released from activated polymorphonuclear neutrophils (Papayannopoulos, 2018). These web-like structures represent a major innate immune mechanism designed to ensnare pathogens (Storisteanu et al., 2017). The surface lipoprotein is a major determinant of NETs formation induced by mycoplasma, and the Toll-like receptor (TLRs) on neutrophil may also be involved in NETs formation. It has been shown that *M. agalactiae* lipoprotein induces the formation of NETs in host cells and is closely related to TLR2 (Cacciotto et al., 2016). Mycoplasma can induce neutrophils aggregating NETs, but it can also avoid NETs. The strategies of pathogens to escape NETs can be divided into three categories: i) NET inhibition via the downregulation of host inflammatory responses; ii) NET degradation through the utilization of pathogen-derived DNases; and iii) NET resistance, which refers to the avoidance of microbicidal components within NETs. These categories can also be referred to as NET inhibition, NET degradation, and NET resistance, respectively (Storisteanu et al., 2017). The strategy of mycoplasma to evade NETs is mainly reflected in the ability to produce nucleases that can reduce NETs. (**Figure 4D**) In those cases, mycoplasma could use the degraded nucleic acid precursors to synthesize substances required for their own growth (Qin et al., 2019). *M. bovis*, *M. agalactia*, *M. pneumoniae*, and *M. hominis* were all found to have corresponding nucleases (Somarajan et al., 2010; Cacciotto et al., 2016; Zhang et al., 2016; Yamamoto et al., 2017; Mitiku et al., 2018; Cacciotto et al., 2019; Li et al., 2019).

Many mycoplasmas secrete nucleases dependent on Ca^2+^ or Mg^2+^. It has been proved that MbovNase from *M. bovis*, encoded by MBOV_RS02825, which is an active Ca^2+^-dependent nuclease and a cytotoxic, secretory protein. It is capable of degrading the DNA matrix of NETs to escape capture and inducing host cell apoptosis (Zhang et al., 2016). Its nuclease activity has been observed to digest linear DNA and RNA from bovine macrophages, as determined by the TNASE_3 region found in the C-terminal of MbovNase, which is similar to the TNASE_3 region derived from a thermonuclease in Staphylococcus aureus (Hynes and Fox, 1991). MHO_0730 is a surface lipoprotein and Ca^2+^-dependent nuclease of *M. hominis*, which could promote NETs formation and degradation. Although it can disrupt the DNA backbone of NETs, it is less efficient in degrading RNA. Its N-terminal lipid moiety could induce the production of Neutrophil extracellular trapsosis (NETosis) in human neutrophils (Cacciotto et al., 2019). NETosis is an inflammatory cell death mode of neutrophils without DNA degradation, which differs from apoptosis and necrosis. And the formation of NETs is accompanied by the death of neutrophils (Papayannopoulos, 2018; Cacciotto et al., 2019). *M. hyopneumoniae* Mhp597, a Ca^2+^ or Mg^2+^ dependent thermostable activity nuclease, can degrade NETs and induce apoptosis. The functional integrity of Mhp597 is unaffected by the absence of its C-terminal 63 amino acids. However, its ability to degrade NETs is compromised in the absence of the enzymatic activity located at the C-terminal site, suggesting a direct correlation between such activity and NETs degradation (Li et al., 2019). While treating *M. bovis* with heat, the formation of NETs was observed. However, live *M. bovis* did not stimulate neutrophil-mediated NETs formation, potentially due to the direct degradation of NETs by the live *M. bovis* nuclease (Gondaira et al., 2021). Some nucleases are produced independently of cations. *M. agalactiae* MAG_5040 has nucleolytic activity that can degrade NETs by digesting their DNA backbone (Cacciotto et al., 2016). Mpn491, the MnuA homologue, a nuclease secreted by *M. pneumoniae*, can disassemble the DNA matrix of NETs to escape the neutrophil elimination mechanism (Yamamoto et al., 2017).

Typically, nucleases can influence DNA stability and enhance their clearance. However, there are exceptions, as Mpn 133 is also a nuclease from *M. pneumoniae* without enzymatic activity, which cannot degrade NETs (Somarajan et al., 2010). As substantiated by existing evidence, nucleases significantly contribute to the breakdown of the DNA matrix of NETs and thus facilitate the immune evasion of mycoplasmas, which can lead to the development of persistent infections and slow-progressing chronic diseases when interacting with the host. Whether mycoplasmas use NET inhibition and NET resistance to degrade NETs remains to be conclusively determined.

### 4.3 Inhibit immune effector molecules

Antibodies and complements are effective immune molecules belonging to the immune system. However, mycoplasma can destroy the antibody through specific mechanisms and escape the killing effect of complement. (**Figure 4E**)

#### 4.3.1 Deal with antibody

It has been demonstrated since 1984 that ureaplasma species possess a serine protease specifically responsible for cleaving the hinge region of IgA1 (Kilian et al., 1984). In contrast to other pathogenic bacteria, such as *Haemophilus influenzae*, *Neisseria meningitidis*, which colonize mucosal surfaces, the IgA1 protease of ureaplasmas is not secreted but rather localized on the cell surface (Arfi et al., 2021). It has been proved that *Mycoplasma synoviae* can digest chicken IgG (cIgG) and express CysP, which can cleave cIgG into Fab and Fc fragments. Indicating that CysP is responsible for the cIgG cleavage caused by *M. synoviae* (Cizelj et al., 2011).

Grover et al. have recently identified a novel 62-kDa surface protein, known as “protein M,” encoded by the MG281 locus of *M. genitalium*. This protein has demonstrated a remarkable capacity to bind with high affinity to the light chains of different immunoglobulins (IgG, IgA, and IgM), thereby inhibiting the formation of immune complexes (Grover et al., 2014). Notably, this ability was previously undocumented in other bacterial species. Hence, the discovery of protein M presents a novel and promising avenue for the development of strategies utilizing immunoglobulin-binding proteins for the manipulation of the immune system, beyond conventional proteolytic antibody degradation mechanisms.

Studies have also revealed the presence of mycoplasma Ig-binding protein (MIB) and mycoplasma Ig protease (MIP), which are encoded by tandemly arranged genes and commonly found in many pathogenic mycoplasmas, often in multiple copies (Arfi et al., 2021). Genes encoding the MIB-MIP system are restricted to mycoplasmas and are disseminated by horizontal gene transfer. These gene products target host immunoglobulins, and they are essential for mycoplasma immune evasion (Arfi et al., 2016). The MIB of *M. pneumoniae*, named MPN400, is a cell surface locator protein that strongly binds to human IgG, IgA, and IgM with an affinity for Plg and Fn, which can fend off immunoglobulins of hosts for immune evasion (Blötz et al., 2020). MPN400 exhibits important abilities to subvert the host immune system, and its potential immunomodulatory effects remain to be elucidated (Blötz et al., 2020).

#### 4.3.2 Inhibite complement activate

In addition to clear antibody, mycoplasma can also escape immune response by binding factor H to escape complement killing. Factor H is a negative regulator of the complement system, which binds to host cells to avoid accidental complement activation. *M. hyopneumoniae* binds factor H via factor H binding proteins, such as EF-Tu, which further contributes to reducing C3 deposition on the surface of *M. hyopneumoniae* and ultimately blocks further complement activation. Also, factor H utilized by *M. hyopneumoniae* can partly increase the adhesion of *M. hyopneumoniae* to swine tracheal epithelial cells through EF-Tu (Yu et al., 2020). In addition, mycoplasma antigen variation can also enable mycoplasma to evade the hunting effect of antibodies, and the mechanism of it will be mentioned below.

In a word, there is a complex interaction between mycoplasma and immune cells, which can inhibit the activity of immune cells, destroy the function of immune cells (avoid phagocytosis and degradate NETs), and inhibit immune effector molecules.

## 5 Antigenic variation

Antigenic variation permeates the majority of the immune evasion system in mycoplasma, serving as a mechanism to evade both phagocytosis (**Figure 4C**) and recognition by antibodies (**Figure 4E**). Consequently, we will now delve into several established classical mechanisms of antigenic variation in mycoplasma. Antigenic variability refers to the capacity of microorganisms to modify their surface components, including flagella, microvilli, membrane proteins etc., leading to different immune response in host (Razin et al., 1998; Betlach et al., 2019). For mycoplasma, its antigens primarily consist of surface-located adhesin, membrane lipoproteins, and related virulence molecules. Variation in the surface antigens of mycoplasma can disrupt the recognition of immune cell and immune molecule. Therefore, it is crucial to investigate the mechanisms underlying antigenic variation in mycoplasma. In this regard, the analysis of mycoplasma antigen variation can be approached from both genomic and proteomic perspectives.

### 5.1 The genomic level illustrates antigenic variation

#### 5.1.1 ON/OFF switching

The high frequency of major antigenic changes within clonal populations of mycoplasma is attributed to the ON/OFF switching mechanism (Citti et al., 2010; Qin et al., 2019). Several mycoplasmas, including *M. hyorhinis, M. bovis, M. mycoides* subsp. *mycoides* strain, *Mycoplasma pulmonis*, and *M. agalactiae etc.* (Bhugra et al., 1995; Citti and Wise, 1995; Lysnyansky et al., 1996; Persson et al., 2002; Czurda et al., 2017), regulate antigenic variation and phage variation through this switching mechanism. There are three main types of ON/OFF switches identified so far. The first type involves a DNA sliding mechanism, and the antigenic variation is achieved through nucleotide insertions or deletions in simple sequence repeat regions. A notable example is the Vlps protein family of *M. hyorhinis.* The phase variation of Vlp products in *M. hyorhinis* is determined by the transcription of *vlp* genes. Each *vlp* gene is expressed as a distinct transcript, and its expression undergoes frequent ON/OFF switches associated with random insertion or deletion mutations in a homopolymeric tract of adenine residues in the promoter region of all *vlp* genes (Citti and Wise, 1995). The bovine pathogen *Mycoplasma mycoide*s *subsp. mycoides* small colony type (*M. mycoides* SC) have identified a variable surface protein, Vmm. Sequencing the *vmm* gene region in ON and OFF clones revealed that the transcriptional regulation of *Vmm* was associated with dinucleotide insertions or deletions within a repetitive region of the promoter spacer (Persson et al., 2002). The second ON/OFF switching type is mainly caused by rearrangement. Chromosomal rearrangements of murine pathogen *M. pulmonis* occur at high frequency, and these rearrangements regulate the phase-variable expression of the gene cluster (*vsa*), which encodes the variable V-1 surface antigen. Only one *vsa* gene was associated with an expression locus in these gene clusters, and the other *vsa* genes were silently expressed. The gene expression can be regulated by recombining the 5’ region of the expressed gene with the 3’ region of the silenced gene in DNA rearrangement (Bhugra et al., 1995; Simmons et al., 1996). The final ON/OFF switch type is promoter inversion. P35 of *M. penetrans* undergoes a high-frequency ON/OFF phase variation, causing changes in the lipid-associated membrane proteins (LAMPs) profile. Thirty-eight *mpl* genes distributed in three clusters encode P35 family lipoproteins. The expression of each *mpl* gene is driven by promoter inversion, which can cause the high-frequency ON/OFF phase variation. These changes help *M. penetrans* escape the immune response of the host (Horino et al., 2003; Distelhorst et al., 2017).

#### 5.1.2 Other variation

Other antigenic variations like size variation and domain shuffling are also essential for mycoplasma. The Vlps protein of *M. hyorhini* mentioned above also undergoes size variation, which is achieved through the spontaneous insertion/deletion of repetitive DNA sequences in the III region of the *vlp* gene (Citti and Wise, 1995). The Vaa size variation of *M. hominis* can affect the adhesion of *M. hominis* and contribute to the evasion of the antibody-mediated humoral immune response (Saadat et al., 2018). *M. genitalium* can transfer DNA horizontally by using a novel mechanism that requires the protein RecA and is facilitated by alternative overexpressed sigma factor σ20, which may accelerate the dissemination of successful antigenic variants within the population (Torres-Puig et al., 2018). The MG428 protein can markedly upregulate the transcription of recA, ruvA, and ruvB, triggering mgpB and mgpC gene variation in *M. genitalium* and ultimately causing antigenic variation and evasion of the host immune system (Burgos and Totten, 2014; Torres-Puig et al., 2015; Burgos et al., 2018). In summary, mycoplasmas can generate multiple antigen variations, which are critical adaptive strategies, enabling organisms to escape host immune defenses and adapt to the host environment at different stages of infection.

#### 5.1.3 Mutiple mechanism contribute to antigenic variation

Based on the discussion above, we have provided an overview of the current academic understanding of the phenotypes and underlying mechanisms of antigenic variation in mycoplasmas. In fact, the variation of antigen on mycoplasma surfaces arises from the collective action of multiple mechanisms. The generation of repeat sequences often coincides with recombination events in mycoplasma antigen variation. The main antigen of *M. pneumoniae*, P1 adhesin, P40, and P90 proteins are responsible for binding host receptors (Vizarraga et al., 2020). The *M. pneumoniae* genome harbors repeated regions, denominated RepMPs. The majority (75%) of it has homology with MPN141 (P1) and MPN142 (P40/P90). Through homologous recombination between RepMPs and MPN141 or MPN142, the variability within antigenic regions of P1 and P40/P90 is generated, which may contribute to the immune evasion strategies of *M. pneumoniae* (Spuesens et al., 2011; Vizarraga et al., 2020). This is compatible with the hypothesis that recombination between repetitive elements may enable *M. pneumoniae* to evade the immune system (Lluch-Senar et al., 2015). Variations caused by repetitive sequences and recombination also take place in *M. genitalium* (Iverson-Cabral et al., 2007; Ma et al., 2015). Multiple antigen peptides containing the mimic epitopes of *M. genitalium* adhesion protein (MgPa) can induce strong immune responses (Zeng et al., 2012; Zeng et al., 2013), and its genes are highly variable. The variation in the MgPa operon has been demonstrated through the recombination of repetitive chromosomal sequences with MG191 (mgpB) and MG192 (mgpC) genes (Ma et al., 2015). While mycoplasmas possess minimal genomic sequences, it is intriguing that they exhibit antigenic variation through multiple genomic mechanisms to escape immune surveillance. This observation underscores the importance of investigating the immune evasion mechanisms in mycoplasmas, as it provides a solid theoretical basis for understanding their pathogenic mechanisms and immune escape strategy.

### 5.2 Post-transcriptional modification

Post-translational modifications belong to proteomics and transcriptomics variability. Protein cleavage events are one of the post-translational modifications, which have been reported in adhesins, lipoproteins, and surface moonlight proteins in *M. hyopneumoniae* (Raymond et al., 2015; Betlach et al., 2019; Li et al., 2020). It was previously reported that the P97 and P102 adhesin families, which undergo post-translational processing, are vital proteins to mediate the attachment of *M. hyopneumoniae* to epithelial cilia (Tacchi et al., 2016). Moreover, most of them are processed highly efficient cleavage events typically at S/T-X-F↓-X-D/E sites (Tacchi et al., 2016). In addition, the differences of the other four most abundant adhesins including (p216) and the homologous of adhesion-related surface proteins (DnaK, p46, and ABC transporter xylose-binding lipoprotein) in the protein hydrolysis process of *M. hyopneumoniae* 7448 (pathogenic) and J (non-pathogenic) were analyzed by LC-MS/MS. Only a few common conserved cleavage sites were found in these homologous proteins. Different cleavage sites lead to protein diversity and may contribute to virulence and pathogenicity (MaChado et al., 2020). Proteomic analysis of translated protein processing events in *M. hyopneumoniae* can expand protein function and expose more epitopes, which may increase the immune burden of the host (Li et al., 2020). There are two distinct forms of the extensively produced macrophage-activating lipopeptide (MALP) product that undergo lipid modification on the surface of *M. fermentans*, of which forms include: (i) the complete, mature product known as MALP-404 and (ii) the lipopeptide MALP-2, containing the N-terminal 14 residues of the mature lipoprotein and activating macrophages through Toll-like receptor 2 (TLR2), and their ratios show significant variation among different isolates. The usage of the detergent phase fractionation technique to isolate cell-bound products and the utilization of N-terminal sequencing for a newly discovered released fragment (RF) revealed that there was site-specific proteolysis between residues 14 and 15 of the mature lipoprotein MALP-404. This resulted in the formation of both cell-bound MALP-2 and soluble RF products (Davis and Wise, 2002). LC-MS/MS was also utilized to analyze the proteins derived from *M. pneumoniae* M129. This analysis successfully identified 22 proteoforms of P1 through terminomics techniques, which allowed for the identification of 17 cleavage events. These findings were further confirmed through proteome studies and immunoblotting assays (Widjaja et al., 2020).

In addition to cleavage modifications, phosphorylation is another important modification that occurs in mycoplasma. Research has indicated that PRKC-associated mutants of *M. pneumoniae* exhibit a loss of cytotoxicity and non-adherent growth. The absence of serine/threonine protein kinase C, encoded by this gene, can disrupt the phosphorylation of cellular adhesion proteins. PRKC not only influences the phosphorylation status of these adhesion proteins but also affects their accumulation within the cell (Goyal et al., 2010). Phosphorylation has been found to have a broader impact on post-transcriptional regulation in *M. pneumoniae* than previously acknowledged, and it may contribute to changes in protein abundance (van Noort et al., 2012). In conclusion, post-translational modification may facilitate mycoplasma immune escape by providing diversity to mycoplasma lipoproteins, which burden the host immune system.

## 6 Molecular mimicry

If the pathogen shares the same substance with the host cell, it may enable the pathogen to evade the surveillance of immune cells. Molecular mimicry is a mechanism through which various sources of infection or other exogenous substances may trigger an immune response to autoantigens due to the similar substance. Anti-GM1 antibodies induced by molecular mimicry in *M. pneumoniae* may cause acute motor axonal neuropathy (Susuki et al., 2004). *M. pneumonia*-specific IgG antibodies can also cross-react with the myelin glycolipid galactocerebroside and cause neurological disorders, including Guillain-Barré syndrome and encephalitis, which constitute the most common and severe neurological extrapulmonary manifestations of *M. pneumoniae* (Meyer Sauteur et al., 2019). Without antibodies, the compensatory immune responses of both innate and adaptive immune cells fail to clear *M. pneumoniae*. The glycolipid subfractions of *M. pneumoniae* are highly immunogenic in mice and humans, display similarity with mammalian tissue compounds, which may trigger multiple cross-reactive antibodies targeting multiple organ system cells of the host (Meyer Sauteur et al., 2019). In the case of *M. pneumoniae*, the P1 adhesin protein has functional sites that exhibit antigenic mimicry with eukaryotic structures. This mimicry may play a role in the pathogenesis of *M. pneumoniae* infection. Furthermore, due to the antigenic similarities between *M. pneumoniae* molecules and human GAPDH and enolase, the immune response during natural infection with *M. pneumoniae* may be non-responsive or self-limited. Patients with primary biliary cirrhosis (PBC) exhibit significantly enhanced frequency of mpPDC-E2-related antibodies, suggesting that molecular mimicry between the surface molecules of *M. pneumoniae* and epitopes of the autoantigen may play a vital role in the etiopathology of PBC (Berg et al., 2009).

The expression of the epitope recognized by murine monoclonal antibodies PF/2A is highly restricted to tumors. However, antigen mimicry may exist between *M. hyorhinis* epitope and a non-blood group, tumor-associated epitope, which may make it act as the pathogenesis adhesion (Fernsten et al., 1987). Due to similar structures between some microbial antigens and the host, specific antibodies or effector T cells against microbial antigens may react with corresponding host antigens and cause autoimmune diseases. In summary, molecular mimicry is part of the immune escape mechanisms of microorganisms.

## 7 Conclusion and perspective

This review summarizes the strategy of mycoplasma for suppressing the host immune response and avoiding detection by the immune system. The cell invasion, making mycoplasma difficult for immune cells to recognize and clear. Mycoplasma can also penetrate epithelial barriers, degrade ECM and travel to other organs, causing chronic infections. Mycoplasma also interacts with different immune cell types, including monocytes, macrophages, and NK cells, and can degrade NETs. The surface lipoproteins or released toxic substances of mycoplasma can inhibit immune cell activity by promoting apoptosis and inhibiting proliferation. The formation of mycoplasma biofilm reinforces evasion and affects the virulence and survival of the pathogen. Antigenic variation also provides strong support for immune evasion by mycoplasma. The insights into the immune evasion mechanisms provide a foundation for developing diagnostics, treatment, and prevention measures against mycoplasma infections.

Cell invasion is undoubtedly a major mechanism employed by mycoplasma to evade immune responses. However, due to the lack of stable cell invasion models and the limited reproducibility of results across different laboratories, there have been relatively few studies on the pathways associated with cell invasion. We have discussed the hypothetical model of mycoplasmas to invade host cells. The ECM, as a bridge between cells and pathogens, plays an important role in this process. Fn has been extensively studied in the context of mycoplasma research. Other components also play significant roles in mediating pathogen invasion. For instance, vitronectin (Vn) can facilitate the interaction between pathogens and integrins, thereby promoting invasion (Singh et al., 2010; Tan et al., 2017), but whether it is related to mycoplasma cell invasion remained to be invested.

In addition, as parasitism, the energy metabolism of mycoplasma intracellular is also worthily investigated. Chlamydia, along with mycoplasma, is a primary cause of lower reproductive tract infections in women. Similar to mycoplasma, chlamydia, being a prokaryote, also relies on residing within living cells and employs comparable methods of cell invasion. This process involves the utilization of adhesins, inducing cytoskeletal changes, and the activation of caveolin-mediated endocytosis, membrane rafts, or clathrin-mediated endosome formation (Mehlitz and Rudel, 2013). However, there are differences in the survival strategies employed by chlamydia and mycoplasma within the host cell. Chlamydia possesses two distinct morphologic types: a highly infectious, non-replicative, small form called the elementary body (EB) and a larger, non-infectious, replicative form known as the reticulate body (RB). Upon infection, EBs are enclosed within membrane-bordered vacuoles referred to as intracytoplasmic inclusions, where they differentiate into metabolically active RBs. RBs utilize host cytoplasmic nutrients and undergo repeated replications via binary fission during the middle phase of the developmental cycle. The transformation of RBs back into EBs occurs when the inclusion containing RBs reaches a critical volume and experiences nutrient and ATP depletion. The newly formed EBs are then released into the extracellular environment, initiating another round of infection (Scurtu et al., 2022). However, similar to mycoplasma, limited research has been conducted exploring the energy metabolism of chlamydia within the host cell. As technology advances, future opportunities may arise for a deeper understanding of the intracellular energy metabolism of these host bacteria.

The interaction between mycoplasma and immune cells is also crucial for immune evasion. Upon infection, mycoplasma and associated virulence factors can activate immune cells, inducing the release of inflammatory cytokines, which also recruit more immune cells to the site of infection for pathogen clearance. However, mycoplasma possesses a series of potent countermeasures to combat immune cells. Surface proteins of mycoplasma exhibit cellular toxicity, which can suppress immune cell activity. Intense inflammatory responses can impair immune cells and cause damage to host tissues and organs. In order to prevent further immune-mediated damage, the host may initiate an anti-inflammatory response. It may allow mycoplasma to persist in the host for a long time, which is not conducive to the elimination of mycoplasma. It is possible that the same substance produced by mycoplasma may have different effects on different cell lines. In a word, the impact of mycoplasma on host immune cells is a complex process that warrants further investigation.

Mycoplasma can evade phagocytosis, break down NETs, and disrupt specific effector molecules. Our study primarily focuses on the mechanisms employed by mycoplasma to combat NETs, predominantly through the degradation of NETs. Correspondingly, various species within *Group A Streptococcus*, such as *Streptococcus pyogenes*, *Streptococcus pneumoniae*, and *Streptococcus suis serotype 2*, produce the nucleases Sda1, EndA, and SsnA, respectively, which result in the breakdown of NETs and facilitate their dissemination (Ríos-López et al., 2021). However, mycoplasma undermines NETs with the intention of not only evading capture but also harnessing phosphoric acid residues present in the degraded DNA structure to ensure its survival and facilitate replication. It coincidence aligns with the multi-drug resistant bacteria, *Pseudomonas aeruginosa*, which also exhibits comparable utilization of NETs degradation products. It have demonstrated that *P. aeruginosa* biofilms enhance the release of NETs and incorporate the released DNA into their polysaccharide matrix *in vitro*. This incorporation results in the tolerance to antimicrobial peptides embedded in the DNA lattice (Rybtke et al., 2015). Additionally, various pathogens like *S. pneumoniae* have the ability to inhibit NETs production. The streptolysin O (SLO) enzyme, produced by *S. pneumoniae*, can hinder several neutrophil functions, including respiratory bursts, degranulation, and extracellular trap formation (Uchiyama et al., 2015). However, it appears that mycoplasma does not employ this particular strategy.and some of it even stimulate NETs production without the presence of ROS (the main product of respiratory burst), such as the *M. bovis* membrane nuclease, Mnua (Mitiku et al., 2018). It is challenging to determine if mycoplasma’s strategy against NETs is intentional.

Antigenic variation in mycoplasmas represents a pivotal mechanism for immune evasion, and genomics and proteomics have shed light on the underlying mechanisms. While mycoplasmas exhibit significant variability in many surface proteins, they also harbor conserved proteins. Even highly variable proteins may contain conserved epitopes or regions. These can serve as a basis for developing mycoplasma vaccines, aiding in the early prevention of mycoplasma infections. The *M. pneumoniae* adhesion capabilities are significantly reduced by polyclonal antibodies generated against conserved C-terminal domain constructs of P1 (Vizarraga et al., 2020). DNA vaccine encompassing amino acid residues 1125–1359 of the C-terminal region (P1C) of the *M. pneumoniae* P1 protein demonstrated discernible protective effects against *M. pneumoniae* infection in BALB/c mice. Notably, there were significant increases in the levels of IgG (including IgG1, IgG2a, and IgG2b isotypes) as well as cytokines (such as IFN-γ and IL-4) (Zhu et al., 2012). Mh128 is a specific, conserved, and immuno-reactive chimeric protein that can be potentially used in immunoassays for diagnosis of *M. hominis* infection in humans and may serve as a suitable antigen for a vaccine against this bacterium (Saadat et al., 2018). With the advancement of technological approaches, future endeavors necessitate the discovery of additional conserved epitopes in mycoplasma proteins, facilitating the development of vaccines to combat infections.

## Author contributions

JH conceived and provided the main direction of this manuscript. JW and KL drafted the manuscript. LC and XS modified the manuscript. DL and JY assisted in the preparation of format. All authors contributed to the article and approved the submitted version.
